# Measuring improvement in knowledge of drug policy reforms following a police education program in Tijuana, Mexico

**DOI:** 10.1186/s12954-017-0198-2

**Published:** 2017-11-08

**Authors:** J. Arredondo, S. A. Strathdee, J. Cepeda, D. Abramovitz, I. Artamonova, E. Clairgue, E. Bustamante, M. L. Mittal, T. Rocha, A. Bañuelos, H. O. Olivarria, M. Morales, G. Rangel, C. Magis, L. Beletsky

**Affiliations:** 1 Division of Global Public Health, UC San Diego – School of Medicine, Third Floor, CRSF, La Jolla, San Diego, USA; 2 San Diego State University, San Diego, CA USA; 3 Facultad de Medicina, Universidad Xochicalco, Tijuana, Mexico; 4 Secretaría de Seguridad Pública Municipal, Dirección de Planeación y Proyectos Estratégicos, Tijuana, Mexico; 5 Secretaría de Seguridad Pública Municipal, Instituto de Capacitación y Adiestramiento Profesional (ICAP), Tijuana, Mexico; 6 Comisión de Salud Fronteriza, México-Estados Unidos, Tijuana, Mexico; 7 Centro Nacional para la Prevención y el Control del VIH y el SIDA (Censida), Mexico City, Mexico; 8 School of Law and Bouvé College of Health Sciences, Northeastern University, Boston, USA

**Keywords:** Police, Harm reduction, Decriminalization, Drug policy, Narcomenudeo, Mexico

## Abstract

**Background:**

Mexico’s 2009 “narcomenudeo reform” decriminalized small amounts of drugs, shifting some drug law enforcement to the states and mandating drug treatment diversion instead of incarceration. Data from Tijuana suggested limited implementation of this harm reduction-oriented policy. We studied whether a police education program (PEP) improved officers’ drug and syringe policy knowledge, and aimed to identify participant characteristics associated with improvement of drug policy knowledge.

**Methods:**

Pre- and post-training surveys were self-administered by municipal police officers to measure legal knowledge. Training impact was assessed through matched paired nominal data using McNemar’s tests. Multivariable logistic regression was used to identify predictors of improved legal knowledge, as measured by officers’ ability to identify conceptual legal provisions related to syringe possession and thresholds of drugs covered under the reform.

**Results:**

Of 1750 respondents comparing pre- versus post training, officers reported significant improvement (*p* < 0.001) in their technical understanding of syringe possession (56 to 91%) and drug amounts decriminalized, including marijuana (9 to 52%), heroin (8 to 71%), and methamphetamine (7 to 70%). The training was associated with even greater success in improving conceptual legal knowledge for syringe possession (67 to 96%) (*p* < 0.001), marijuana (16 to 91%), heroin (11 to 91%), and methamphetamine (11 to 89%). In multivariable modeling, those with at least a high school education were more likely to exhibit improvement of conceptual legal knowledge of syringe possession (adjusted odds ratio [aOR] 2.6, 95% CI 1.4–3.2) and decriminalization for heroin (aOR 2.7, 95% CI 1.3–4.3), methamphetamine (aOR 2.2, 95% CI 1.4–3.2), and marijuana (aOR 2.5, 95% CI 1.6–4).

**Conclusions:**

Drug policy reform is often necessary, but not sufficient to achieve public health goals because of gaps in translating formal laws to policing practice. To close such gaps, PEP initiatives bundling occupational safety information with relevant legal content demonstrate clear promise. Our findings underscore additional efforts needed to raise technical knowledge of the law among personnel tasked with its enforcement. Police professionalization, including minimum educational standards, appear critical for aligning policing with harm reduction goals.

## Background

The predominant framework for drug policies around the world focuses on criminalization and punishment [1]. Meanwhile, this legal framework has also unintended collateral harm, including street violence, occupational risks to police, police corruption, spread of infectious diseases, and various social and economic detriments [2]. Mexico’s drug war has claimed the lives of more than 1000 police officers [3], and its toll on civilians is in the hundreds of thousands [4, 5]. In the realm of infectious disease, arbitrary policing tactics such as syringe confiscation have been associated with higher risks of human immunodeficiency virus (HIV) transmission [6]. Incarceration of drug users increases their risk for blood borne infections, due to high levels of drug use, scarce access to injection equipment and condoms, and exposure to sexual abuse and extortion [7, 8]. Amidst regional efforts to reduce harms emanating from this punitive framework, police enforcement, either through formal or informal practices, should be included as factors shaping public health outcomes [9, 10].

In Mexico, drug production and trafficking intended for the USA has expanded to a domestic market, where “narcomenudeo” (retail drug sale) functions as an additional source of revenue for the drug cartels [11]. To meet these challenges, the federal government moved to involve local authorities in the prosecution of minor drug crimes. In the context of drug policy reforms elsewhere in North America, Mexico’s agenda was similarly driven by an increase in drug-related violence [12], overcrowded local jail systems [13], and an international shift toward evidence-based practices that emphasize the necessity to treat drug use as a public health problem rather than as a criminal issue [14].

The 2009, amendment of the General Health Law and Federal Penal Code, commonly known as the “narcomenudeo reform” served as the centerpiece of Mexico’s broader criminal justice reform. These law reforms decriminalized [15–17] possession of small amounts of drugs, while delegating criminal prosecution of retail drug sales down from the federal to the state level [18]. The reform mandated that, when apprehended by police, individuals possessing small quantities of drugs for personal consumption below a specified volume would not be charged with a crime. Instead, these users must be referred to the health authorities and then released, avoiding jail time altogether. However, in the third time an individual is caught with drugs under the specified threshold, the “narcomenudeo reform” mandated them to enter drug treatment provided by the state [17]. The federal law additionally set a deadline of August 2012 for full implementation and funding of the reforms on the local level. All states had to modify their penal codes and local regulations, such as police procedures and treatment referrals, to comply with the new regulations [18]. The “narcomenudeo reform” adopted a public health-based approach to drug use by promoting treatment and harm reduction rather than incarceration. By decriminalizing possession of small amounts of drugs, it held the potential to reduce criminalization of users, instead facilitating their engagement with substance use treatment services. These reforms were also supported by broader efforts to professionalize law enforcement and the judicial system in Mexico to improve the administration of justice, including the creation of federal guidelines for minimum education standards for municipal police [19]. Despite the significance and controversy surrounding these professionalization efforts, their impact on drug policy reform efforts has never been formally evaluated.

The city of Tijuana is an important setting for evaluating the implementation of the “narcomenudeo reform” as it experiences a disproportionate burden of drug-related harms. Located in Baja California on the northwest Mexican border with the USA, the city forms an urban border region and is the busiest land border crossing of the world. As a result of active north- and south-bound migration, the city is home to large numbers of high-risk individuals such as sex workers, people who inject drugs (PWID), and deportees from the USA [20, 21]. The city is a major route for drug trafficking and consumption of heroin, cocaine, and methamphetamine [22].

Compared with the national average in Mexico (0.2%) [23], Tijuana also has one of the highest rates of per capita of injection drug use [22], resulting in high prevalence among PWID of hepatitis (95%) C virus (HCV), latent TB infection (57%) [24], and HIV (3.5% among male PWID and 10% among female PWID) [25, 26]. Of concern is the possibility that HIV and other sexual transmitted diseases are likely to spread to other vulnerable populations in close proximity in the city [27]. Access to evidence-based drug treatment and other harm reduction services prior to the implementation of “narcomenudeo reform” were low [28, 29]. Previous research by our team had identified syringe confiscation by police officers to be independently associated to an increase on receptive needle sharing and a higher prevalence of HIV infection among PWID in the city of Tijuana [30], although it is legal to carry syringes without a prescription in Mexico. The new reforms do not specifically address the possession of syringes containing drugs. Our studies regarding needle-stick injuries among police in Tijuana have found that a substantial proportion of officers report regularly encountering syringes, including syringes that contain drugs [31]. Drug decriminalization, reduction of syringe confiscation, and scale-up in access to substance use treatment in Tijuana have the potential to reduce both local and regional harms related to drug use. However, a lack of legal knowledge among those charged with the law’s enforcement can undermine the public health goals of the “narcomenudeo reform,” reducing access to treatment or generating unnecessarily incarceration.

Our prior research among PWID in Tijuana indicated that the implementation of the “narcomenudeo reform” has been limited. Drug user experiences suggested apparent gaps in police knowledge of drug possession laws and related policies [32–34], echoing a growing evidence base of gaps in police knowledge of drug policies [35, 36]. In our mixed-methods cohort study of PWID, conducted between 2010 and 2013 in the city of Tijuana, drug users reported near-total absence of experiences linked to the operational components of the reform [32]. Other observers have expressed concerns that the reform may inadvertently intensify criminalization of users [37]. This is because suspects possessing any drug quantities, including loaded syringes, are theoretically supposed to be presented to the public prosecutor’s office (Ministerio Publico), regardless of police field assessment [37].

To improve street-level implementation of the law, we designed a police education program (PEP). The PEP follows an occupational safety framing in covering drug possession, legality of syringe possession and other formal laws, enforcement procedure, and the rationale for public health initiatives targeting drug users (Project ESCUDO [SHIELD]) [38]. Providing street-level officers with this information, along with content regarding the rationale for, and potential benefits of the reforms could shift police practices toward harm reduction goals contemplated by the “narcomenudeo reform” [39].

The objectives of this study were to determine if the PEP was associated with improvements in police officers’ self-reported knowledge of Mexico’s drug and syringe possession laws. In order to inform future training and other structural interventions, we also sought to identify officer characteristics associated with improved knowledge of drug policy content under the PEP.

## Methods

### Study setting

The Tijuana municipal police force is among the largest in Mexico, with an estimated 2000 officers, divided among 11 policing precincts. These precincts are drawn similarly to match the geographical boundaries of the political-administrative divisions within the municipality. Officers are equally distributed among the precincts, and they are expected to periodically rotate from one to another. Candidates for the police force are mandated to meet a minimum set of requirements, including 3 years of residency in the state, at least a high school education, being aged 18 or older, and passing a set of toxicological and polygraph examinations. The minimum educational requirement was instituted in 2008, but about 20% of existing officers who did not have a high school diploma were grandfathered in, meaning they were exempt from the new regulation and able to continue working in the Tijuana police department. Since 2007, the average annual turnover has been relatively low, at 6.2%, and the police salary is among the highest in the country. The average age of police officers in the force is 38 years old and predominantly (80%) male. Due to federal support designed to incentivize police professionalization, annual refresher training consisting of various modules is required [40].

### Instructional design

The design and conceptual framework of Proyecto ESCUDO have been described elsewhere [38]. Briefly, in 2013, we established a Memorandum of Understanding with Tijuana Ministry of Public Safety (SSPM) to collaborate on police training and assessment activities. While the program was initially designed as a stepped-wedge randomized controlled trial, subsequent policy and programmatic imperatives at SSPM complicated random assignment. However, the design still contains core elements of a stepped wedge design, where pre-intervention period for each officer is treated as the control. The PEP was integrated to the department’s annual training effort, and each week, a cluster of police officers (20–50) from different ranks and precincts received the training. The program covered almost 85% of the total police force within 1 year, and each session lasted approximately three and a half hours in average.

To improve the implementation of the “narcomenudeo reform,” the training featured three modules on relevant drug policies. These modules contained legal information about the drug quantities decriminalized for personal possession and a description of public health-based interventions targeting PWID in the city, such as syringe exchange programs (SEP) and opioid substitution treatment (OST); all of this reinforced through visual aids resembling threshold volumes of specific drugs and video vignettes to help in knowledge acquisition. Within its overarching occupational safety framework, the PEP included content emphasizing the legality of syringe possession by PWIDs and the application of this provision to needle stick injury prevention among police [38, 41]. Specifically, the training underscored that officers should communicate the legality of syringe possession to suspects before a pat-down search, thereby encouraging individuals to volunteer syringe possession and discouraging unauthorized syringe confiscation. This doctrinal content was reinforced through an interactive role-play exercise.

Data presented in this manuscript are drawn from the pre- and post-training evaluations, where the assessments were made immediately before and immediately following the intervention and were limited to those who completed pre- and post-self-administered paper surveys. The questionnaire was adapted from instruments used in previous PEP evaluations in the USA and elsewhere [42]. Bilingual staff translated and back-translated the survey from English to Spanish; trained interviewers piloted the instrument for cultural appropriateness, clarity, and other elements with six officers from the Tijuana police academy—ICAP (*Instituto de Capacitacion y Adiestramiento Profesional*). The survey included socio-demographics (age, gender, education, marital status, rank, years of service), basic infectious disease knowledge, legal knowledge including provisions of the “narcomenudeo reform” (e.g., decriminalized personal consumption quantities), other relevant policies like the legal status of syringes, and attitudes toward harm reduction programs.

### Data collection

Police officers who provided written consent to participate in the evaluation of Proyecto ESCUDO were given self-administered paper surveys (15–20 min to complete) immediately before and after the training. Unique identifiers were generated for each participating officer, which we used to match pre- and post-training data. Officers received compensation (movie tickets worth approximately 20 USD) for the completion of both surveys. All information was confidential, and participants were informed that there were no consequences of their decision to participate on their current or future employment within the SSPM Tijuana.

### Measures

Respondent technical legal knowledge was measured by correct recall of the exact quantity for decriminalized under the “narcomenudeo reform” for three most commonly misused drugs [43, 44], heroin (≤ 50 mg), methamphetamine (≤ 40 mg), and marijuana (≤ 5 g), and an additional measure for the legality of syringe possession (as many as they want). For each of the four outcomes, we dichotomized the responses by correct or incorrect quantities under the law.

Police officers might have difficulty identifying precise weight limits (e.g., grams vs. milligrams), especially since substances sold on the black market are variously packaged as balloons, rocks, or joints. Nevertheless, these officers may understand the general concept of drug decriminalization and act on the “spirit” of the law, even if they do not recall the law’s particular technical elements. To measure this important phenomenon, we created a second set of outcomes that captured the understanding of the legal concept of “decriminalization” [15, 16] for the three substances of interest, in addition to the possession of an unlimited quantity of syringes.

We designated a respondent to have conceptual legal knowledge if, in response to the question about allowable drug volumes and syringes, he/she chose any of the listed quantities. Those choosing options “none” or “do not know” were defined as not having conceptual legal knowledge for each of the four items of interest. To understand the measurement of change in this outcome, we excluded those participants who demonstrated conceptual legal knowledge at baseline: heroin (*n* = 197), methamphetamine (*n* = 201), marijuana (*n* = 283), and syringes (*n* = 1111). Within this sub-sample, we defined as “learners” those participants who demonstrated improvement in conceptual legal knowledge as the result of the training, while those who did not were defined as “non-learners” (i.e., answered options “none” or “do not know” in post-PEP survey).

### Statistical analysis

We used McNemar’s test for paired nominal data to evaluate the impact of the training by comparing the percentage of correct answers on legal amounts of the three substances (for both the exact quantities and the concept of decriminalization), before and after the PEP. This test is an appropriate tool for the analysis of pre-post differences in dichotomous items, as it controls for the individual characteristics of each paired subject [45], in this case the officers’ socio-demographics characteristics.

We used logistic regression to identify correlates of conceptual legal knowledge acquisition (“learners” versus “non-learners”) after receipt of the training. In univariate analyses, only education was found to be significantly associated with all of the outcomes (*p* ≤ 0.05) after considering the following independent variables: socio-demographics (gender, age, years of service, district, education), occupational safety measures (e.g., needle stick injury history, frequency of syringe contact), and pre-PEP attitudes toward harm reduction and decriminalization laws. However, to avoid a specification error and overestimating the effect of the education variable, we control for age and gender in the multivariable analyses [46].

## Results

Overall, 1806 officers were trained by ESCUDO and 1788 (over 99%) agreed to participate in the evaluation, in total 18 police officers refuse to participate in the study. We were unable to match the survey information for 37 officers, so they were excluded from the analysis. Our final sample was comprised of the 1750 officers for whom we had matched pre- and post-training surveys. Respondents had approximately equal representation from 11 police precincts (see Table 1). While the training included officers from all ranks, the majority were street-level officers (81%). The sample represents mostly men (85.9%), with a mean age of 38.5 years (IQR 32–44) and an average of 11 years of work experience (SD 3.47 years). Even though the police requirements mandate a minimum level of high school, almost 20% of officers (*N* = 321) did not meet this standard. Socio-demographic information for officers who had conceptual legal knowledge at baseline can be found in Table 4 in the Appendix.

**Table 1:** Socio-demographic variables for officers participating in ESCUDO

Variable	Total quantitative (*n* = 1750)	
	*N*	% (IQR)
Socio-demographics		
Gender, male	1499	85.90%
Age, mean (IQR)	38.5	(32–44)
Education (*N* = 1596)		
Less than high school	321	20.11%
High school completed	938	58.77%
more than high school	337	21.12%
Total years in law enforcement, mean (IQR)	11	(8–18)
Rank (*N* = 1749)		
District Chief	17	1%
Deputy	73	4.20%
Supervisor	115	6.60%
Officer	1473	84.20%
Current assignment (patrol)	1410	81%

*IQR* interquartile range

Pre- and post-PEP knowledge outcomes for each of the three substances are presented in Table 2. In the realm of precise technical knowledge, we found statistically significant increases (*p* < 0.001) for all substances associated to their participation in the training. At baseline, only 9.1% of police officers could correctly identify the technical quantities of marijuana, 7.5% for methamphetamine, and 8.4% for heroin decriminalized under the current law. After the training, technical legal knowledge of threshold amounts increased to 70% for heroin and methamphetamine and 52% for marijuana. As for syringe possession, we also found a statistically significant increase (*p* < 0.001) from 56 to 90.5% of precise technical knowledge.

**Table 2:** Changes in technical and conceptual legal knowledge about most commonly misused drugs. This includes matched McNemar’s *χ* ^2^ tests between pre- and post-ESCUDO training (*n* = 1750)

Variable	Before training		After training		*p* value
	No.	%	No.	%	McNemar’s *χ* ^2^
Exact knowledge of the law—what a suspect can currently possess under the law of Baja California					
Syringes (as many as they want)	908	55.91	1470	90.52	< 0.001
Heroin (50 mg)	142	8.42	1194	70.82	< 0.001
Methamphetamine (40 mg)	125	7.46	1144	68.26	< 0.001
Marijuana (5 g)	155	9.18	889	52.67	< 0.001
Decriminalization concept—what a suspect can currently possess (any quantity)					
Syringes	1085	66.81	1556	95.81	< 0.001
Heroin	196	11.63	1541	91.4	< 0.001
Methamphetamine	201	11.99	1495	89.20	< 0.001
Marijuana	282	16.71	1545	91.53	< 0.001

Regarding conceptual legal knowledge, respondents showed even more dramatic improvement (*p* < 0.001). At baseline, between 11.6 and 16.7% of police officers conceptually understood the fact that some amount of heroin, methamphetamine, and marijuana had been decriminalized under the current law, regardless of whether they could identify the precise threshold. Post training, almost 90% of the officers selected some decriminalized amount of all three listed drugs. For marijuana possession, there was a major (38.8%) difference between technical legal knowledge and conceptual legal knowledge, whereas those gaps were smaller for heroin (20.5%) and methamphetamine (20.9%). Notably, a minority of police officers (approximately less than 10%) responded that there were no decriminalized quantities of drugs under the current law even after receiving the training. The difference between legal and conceptual legal knowledge for syringe possession is markedly narrower (5.2%), likely underscoring the importance of framing the legality of syringes as an occupational safety benefit and reinforcing doctrinal knowledge through interactive training elements.

Adjusted odds ratios (aOR) and 95% confidence intervals from multivariable logistic regression models examining predictors for improvement of conceptual legal knowledge acquisition between “learners” and “non-learners” after receipt of the PEP are found in Table 3. Only level of education was significantly associated with conceptual legal knowledge uptake for all of the analyzed substances. Individuals with high school education were at least twice as likely to be learners compared to those with lower education, for syringes (aOR 2.6, 95% CI 1.3–5.2), heroin (aOR 2.7, 95% CI 1.7–4.3), methamphetamine (aOR 2.2, 95% CI 1.4–3.2), and marijuana (aOR 2.5, 95% CI 1.6–4).

**Table 3:** Multivariable logistic regression analysis to identify correlates of conceptual legal knowledge acquisition (“learners” versus “non-learners”) after receipt of the ESCUDO training. This includes adjusted odds ratio (aOR) and 95% CI

Predictor	Syringes	Heroin	Methamphetamine	Marijuana
	aOR	aOR	aOR	aOR
Age	0.99	0.99	1.01	0.99
(Lower 95% CI–upper 95% CI)	(0.96–1.02)	(0.97–1.01)	(0.98–1.02)	(0.97–1.01)
Gender (male reference group)	1.38	1.44	1.11	1.4
(Lower 95% CI–upper 95% CI)	(0.59–3.23)	(0.77–2.70)	(0.67–1.84)	(0.74–2.63)
Education (no-HS reference group)				
High school	2.67***	2.75***	2.21***	2.56***
(Lower 95% CI–upper 95% CI)	(1.37–5.22)	(1.76–4.31)	(1.47–3.29)	(1.64–4.02)
More than high school	2.1*	2.25***	2.35***	2.48***
(Lower 95% CI–upper 95% CI)	(0.89–4.91)	(1.27–3.97)	(1.39–3.39)	(1.36–4.51)
Observations	556	1373	1359	1287

***p < 0.01, **p < 0.05, *p < 0.1

## Discussion

Despite several years since the full implementation of the “narcomenudeo reform” in Baja California, we found dismally low levels of legal knowledge of its decriminalization provisions among police officers in Tijuana. These results underscore the failure of an effective rollout of the reforms in that locale. The PEP effect measured here was associated with a dramatic increase of the participants’ technical and conceptual legal knowledge related to decriminalization for marijuana, heroin, and methamphetamine, as well as the legality of syringe possession. Predictably, officers were somewhat less successful in correctly recalling the law’s precise technical provisions. Our findings suggest that the effort to maintain minimum educational requirements [47] (i.e., high school diploma) may improve the uptake of trainings such as the PEP described here. The use of a standardized test measuring educational performance could also help incentivize improved instructional coverage and learning tools among police officers. International literature reflects growing recognition of the importance of PEPs to inform police practices targeting vulnerable groups [48, 49]. In evaluating the legal knowledge element of the PEP, this study adds to this emerging evidence base and highlights the successes, challenges, and opportunities of harm reduction-focused police instruction [50].

While many policy changes regarding drug policy and public safety in Mexico lack a theory-driven approach, the “narcomenudeo reform” case underscores the need for better empirical understanding of the targeted objectives, operational resources, and a more effective policy evaluation framework that includes public health goals. Our previous analysis suggests that the implementation of operational components of the reform are close to non-existent in Tijuana [32]. Using partial data from freedom of information requests, other authors have concluded that while federal detentions for drug crimes have increased, this phenomenon has not been reflected in trends on the local level. More studies that triangulate information from drug user, police, and judicial sources could help to better evaluate the impact of the reform [51].

Drug policy reform is often necessary, but not sufficient to achieve public health goals because of gaps in translating formal laws to policing practice [52, 53]. To close such gaps, PEP initiatives bundling occupational safety information with relevant legal content demonstrate clear promise. Since the ESCUDO project seeks to close the gap in the implementation of a harm reduction-oriented policy [38, 52], identifying factors that potentiate better uptake of legal information is critical to better roll out of the law.

Evaluation of past police education initiatives, including those focused on harm reduction content, has noted discrepancies in uptake and receptiveness of trainings based on a number of characteristics. These studies have included demographic factors like age, education level, and number of years working as a police officer, as well as attitudes on harm reduction and other occupational safety factors [5]. Our analysis found that only educational level was significantly associated with legal knowledge improvement after adjusting for other socio-demographic factors. In a novel way, this reframes police professionalization efforts such as mandatory employment standards [54] as potentially instrumental for harm reduction. We have found only one previously published study examining predictors of legal knowledge acquisition among officers, which identified years of police service as a significant predictor for follow-up knowledge intake [55]. Our study, with its large sample size and high participation rate, substantially expands the evidence base, with implications for instructional design and tailoring.

Nonetheless, legal knowledge change alone is likely not sufficient to transform drug law enforcement. Structural factors such as deeply ingrained stigma, arrest, and other punitive enforcement incentives and other contributing influences also shape police practices. Nevertheless, it is difficult to see how the harm reduction potential of Mexico’s “narcomenudeo reform” and syringe possession provisions can be harnessed without improving dismal levels of officer knowledge about the black letter law. This alone makes legal knowledge a vital intervention target; specific contribution of legal knowledge, attitudinal, and other training components to behavior change will be explored in future analyses using follow-up cohort data.

Prior police reform in Mexico has shown that local police forces lack the education, resources, and proper accountability controls to do their jobs effectively [56]. In addition, policing strategies might change from administration to administration, in part due to the lack of a strong civil public sector career system which relies more on informal rules or patronage appointments [40]. Efforts to standardize and professionalize policing have taken the form of federal guidelines and subsidies, among others [19]. Street-level officers and management should be involved in planning the reforms, so collaboration with external actors and the police can be successful by combining a rigorous evaluation of the results and adapting them to the realities of policing on a daily basis [56].

The correct implementation of the “narcomenudeo reform” has great potential to shift Mexico’s response to substance use to one rooted in public health rather than in a counterproductively punitive approach. The differentiation of drug possession levels between users and traffickers could theoretically help the criminal justice system to optimize its resources by transitioning from an aggressive police enforcement model to a more harm reduction-oriented approach contemplated by the “narcomenudeo reform.” However, our findings indicate that such policies require additional efforts to assure police compliance with the letter and spirit of the laws. The successful experience of the Portuguese reform could help to motivate police management to embrace drug decriminalization as a way to reduce the burden of drug-related harms on the criminal justice system. Portugal saw a decline in costs associated to the imprisoning of drug offenders, overcrowded jails, and reducing the time officers spent in dealing with drug offenses instead of solving high impact crimes [57].

It is imperative to reconsider the drug volumes deemed reflective of typical personal consumption amounts established by the reform. As demonstrated in other cases around the world, the thresholds for personal possession must be meaningful, closely related to the market realities, and in no situation should they lead to arrest or criminal prosecution [17]. As expressed by other observers, the setting of artificially low threshold quantities might expose casual consumers to excessive criminalization Others have posited additional theoretical unintended harms, such as consumers being incentivized to purchase larger amounts to avoid frequent contact with drug dealers [37]. It is also worth noting that decriminalization schemes vary around the world, and there are many other factors at play other than the threshold quantities, such as the role of medical professionals, the institutional capacity of the judicial sector, or the existing social norms toward drug use, that must be taken into consideration to create an effective model [17].

The foundation of this project was a unique collaboration between an academic institution and a police department, including its training academy. The partnership between UCSD and the SSPM Tijuana, the first study of its kind in Latin America, offers valuable lessons that could shape police training, practices, and drug policy implementation in Mexico and elsewhere across the region and globally. Sustainable collaborations between academic institutions and local governments are critical to long-term strategies for police reform and institutionalizing a synergy between two areas that have traditionally operated in isolation from one another, police and public health.

### Limitations

Our study is subject to several limitations. First, a threat to internal validity might be present if the questions on the survey do not measure the constructs as expected. Although a self-administered survey helped reduced social desirability [58], an interviewer-administered survey would have helped determine if the officers properly understood the questions. However, the costs and logistical difficulties to evaluate a large classroom simultaneously made it unfeasible. Previous studies have documented high level of concordance between these two modes of survey administration [59]. There is also a risk of having a non-response bias, where police officers that refuse to take part in the study might express systematically different answers from those included in the evaluation [60]. In total, 37 (0.72%) officers were excluded due to the inability to match pre- and post surveys and were unable to compare these missing data to the rest of the sample used for the analysis. Further, 18 (0.99%) refused to participate due to concerns with privacy and the impact on their job. Thus, due to the low refusal rate/missing data, we expect the effect of this bias to be negligible. Lastly, the experience of police officers in Tijuana may be different than other cities in Mexico, making it difficult to generalize our findings to other police departments in the country [61].

## Conclusion

As an initial phase of a larger training evaluation effort, this study presents an assessment of the factors shaping police knowledge uptake regarding drug and syringe possession. Efforts to improve police legal knowledge are critical to any drug decriminalization or other harm reduction-focused police intervention. Our findings have special relevance for the regional evolution of harm reduction as federal, state/provincial, and local governments embark on drug policy and other reforms. Such reforms are not self-implementing and must be supported by other interventions designed to improve their street-level impact. Broader police reform and professionalization is an under-recognized structural factor that has the potential of improving the state of harm reduction in North America and beyond.

## Abbreviations

HCV: Hepatitis C virus
HIV: Human immunodeficiency virus
ICAP: Instituto de Capacitación y Adiestramiento Profesional Tijuana
PEP: Police education program
PWID: People who inject drugs
SSPM: Tijuana Ministry of Public Safety
TB: Tuberculosis
UCSD: University of California, San Diego
